# Investigating determinants of yawning in the domestic (*Equus caballus*) and Przewalski (*Equus ferus przewalskii*) horses

**DOI:** 10.1007/s00114-016-1395-7

**Published:** 2016-08-19

**Authors:** Aleksandra Górecka-Bruzda, Carole Fureix, Anne Ouvrard, Marie Bourjade, Martine Hausberger

**Affiliations:** 1 Department of Animal Behaviour, Institute of Genetics and Animal Breeding, Polish Academy of Sciences, Jastrzębiec, Poland; 2 CNRS UMR 6552 Ethologie Animale et Humaine, University of Rennes 1, Rennes, France; 3 School of Veterinary Sciences, University of Bristol, Langford House, Langford, Bristol BS40 5DU UK; 4 UMR CNRS 5263 Cognition Langue, Langage, Ergonomie, Laboratoire Travail et Cognition, University of Toulouse Jean Jaurès, Toulouse, France; 5 Association pour le cheval de Przewalski: TAKH, Station biologique de la Tour du Valat, Arles, France

**Keywords:** Yawning, Domestic horse, Przewalski horse, Sex, Stress, Welfare

## Abstract

Yawning is rare in herbivores which therefore may be an interesting group to disentangle the potential function(s) of yawning behaviour. Horses provide the opportunity to compare not only animals living in different conditions but also wild versus domestic species. Here, we tested three hypotheses by observing both domestic and Przewalski horses living in semi-natural conditions: (i) that domestic horses may show an elevated rate of yawning as a result of the domestication process (or as a result of life conditions), (ii) that individuals experiencing a higher level of social stress would yawn more than individuals with lower social stress and (iii) that males would yawn more often than females. The study involved 19 Przewalski horses (PHs) and 16 domestic horses (DHs) of different breeds living in large outdoor enclosures. The results showed that there was no difference between the PH and DH in yawning frequency (YF). PHs exhibited much higher levels of social interactions than DHs. There was a positive correlation between yawning frequency and aggressive behaviours in PHs, especially males, supporting the idea that yawning may be associated with more excitatory/stressful social situations. A correlation was found between yawning frequency and affiliative behaviours in DHs, which supports the potential relationship between yawning and social context. Finally, the entire males, but not castrated males, showed much higher levels of yawning than females in both species. The intensity (rather than the valence) of the interaction may be important in triggering yawning, which could therefore be a displacement activity that helps reduce tension.

## Introduction

Yawning is a behaviour that is performed by different vertebrate species (Siamese fighting fish, lions and primates, Baenninger 1987; dogs, Dreschel and Granger 2005) including humans (Baenninger 1997). This physical event has been described in detail in humans as being composed of three distinct phases: a long inspiratory phase, a brief acme and a rapid expiration (Baenninger 1997). Yawning was studied in rodents (e.g. Moyaho et al. 1995; Fundaro 1996), carnivores (e.g. Bekoff 1974; Leyhausen 1979; Joly-Mascheroni et al. 2008), birds (Gallup et al. 2009, 2015) and, most extensively, non-human (e.g. Deputte 1994; Hadidian 1980; Troisi et al. 1990) and human (e.g. Baenninger et al. 1996) primates. In non-human primates and rats, this behaviour is more frequent in males (Berendsen and Nickolson 1981; Troisi et al. 1990). According to Smith (1999), yawning is related to changes in the arousal state (i.e. alertness/drowsiness, Baenninger and Greco 1991; Greco et al. 1993), pandiculation (Gessa et al. 1966), thermoregulation and brain cooling (Gallup and Gallup 2007, 2008) and/or can correspond to the expression of social status (Deputte 1978) or stress (Maestripieri et al. 1992; Beerda et al. 2000). It has also been proposed that yawning could, in some contexts, be a type of *displacement* behaviour (Tinbergen 1952; Troisi 2002). In primates, increased yawning frequencies were observed in groups with higher numbers of conflicts, especially in cases of changes in group composition (hence establishing hierarchy and increased social stress), primarily in males. This observation has led to the *social stress-related* hypothesis of yawning (Troisi 2002; Deputte 1994). Deputte (1978) classifies the yawning in *non-human* primates into three types: (1) physiological yawn, related to sleep/awakeness activity; (2) *stress yawn*, occurring around conflict/frustration situations; and (3) threat yawn.

As compared to primates and carnivores, yawning occurs at a lower rate and has been described in a limited number in herbivore species (Baenninger 1997; Gallup 2011) for which the context and potential functions are therefore still less known. Recent research on domestic horses has, however, evidenced a co-occurrence of yawning and stereotypic behaviour in restricted domestic situations, indicating frustration as a potential indirect causal factor for those behavioural patterns (Fureix et al. 2011). Interestingly, the frequency of occurrence of this behaviour was rather high (around 2 per h) and did not differ between sexes which opposes studies on other mammalian species (e.g. Holmgren et al. 1980; Troisi et al. 1990). However, with regard to sex differences, only geldings (castrated males) and mares were involved in the study of Fureix et al. (2011). The authors argued that the restricted housing conditions may be responsible for this rather high frequency of yawning, but since no research had been performed on horses living in natural conditions, this was difficult to assess. Additionally, it has been proposed that domestication, due to selection of animals, may change the frequency of occurrence of particular behaviours, like, for instance, the decrease in territorial competition or exaggerated reproductive behaviour in domesticated species (Price 1999). Thus, the idea that domestication could have promoted more frequent yawning may be further investigated by comparing the wild species (*Equus przewalskii*) to the domesticated species, in both cases within socially stable groups of animals living in semi-natural conditions.

The aim of the present study, therefore, was triple: (i) testing the potential effects of domestication by examining the frequency of yawning in semi-natural conditions in wild (*Equus ferus przewalskii*) and domesticated (*Equus caballus*) species (and, secondarily, of life conditions by comparing with published data in restricted conditions of life); (ii) to some extent, testing the social stress hypothesis proposed by Deputte (1994) by exploring the possible association between yawning and social interactions, in particular agonistic behaviour frequencies; and (iii) assessing sex differences in yawning frequency in both species by comparing males and females. Since testosterone has been shown to be involved in yawning frequency in other species and males being more often involved in social rank competition, we hypothesised that individuals (and potentially males) in groups experiencing a higher level of social stress would yawn more. The horse, having different social structures from harems to bachelor-only groups, is an interesting *non-primate* mammal species to investigate further potential determinants of yawning. Also, yawning is considered to be an extremely rare behaviour in ungulates (Gallup 2011). The latter, as contradicted by the study of Fureix et al. (2011), was then studied in two species of Equidae: Przewalski and domestic horses.

## Material and methods

### Subjects

Przewalski (*E. ferus przewalskii*) and domestic (*E. caballus*) horses were used in the study. For the Przewalski horses, the data obtained in the context of a former study (Bourjade 2007) have been reanalysed in order to look more precisely at yawning behaviour.

The Przewalski horses (PHs, *N* = 19) had been observed during two periods (on May and June 2004 and on April and May 2005) at ‘Le Villaret’ (France), where they are kept in a large 280-ha enclosure. Because they are involved in a reintroduction program, the horses are left with a minimum of human interference (only some hay in harsh winters); groups have formed naturally and were moving freely for vital resources within this large enclosure. Behavioural data were available for adult males (*N* = 8) from two bachelor groups (composed of four to six non-reproductive adult males) and for 1- and 2-year-old male (*N* = 5) and female (*N* = 6) horses from five independent harems.

Two populations of domestic horses (DHs) were observed on March and April 2008: the first population was located near the Paimpont Biological Station of University of Rennes 1, France, and composed of seven horses of varied breeds (warmbloods, ponies and their crosses), distributed into two *bachelor* groups (group 1 consisting of two males and three geldings (castrated males) and group 2 consisting of two males), and the second population was located at the Research Station of Ecological Agriculture and Breeding of Endangered Animals, Polish Academy of Sciences in Popielno, Poland, and composed of two groups: group 1 consisting of one adult male with seven females and their offspring (foals; three individuals) and group 2 consisting of one male with three females and two foals, all of Konik polski breed (*N* = 12). Given the small number of domestic stallions in each type of social group, for data analysis, the calculations were made on the total number of mature male DHs (all DHs, *N* = 6).

All horses (PH and DH) lived in stable groups in large outdoor enclosures (from 2 to 1600 ha), and the adult horses had been in this same group for at least 2 years. All domestic horses had free access to natural feed and water resources and were supplied with hay only in harsh winters.

### Methods

The animals were under the care of the staff of the Popielno Research Station in Poland, the Association Takh in Le Villaret, France, and a private owner in Paimpont, France. The animals, observed in the conditions close to natural, were enabled to express a free interaction with their group mates.

### Behavioural observations

Each horse was observed using a focal continuous sampling method (Altmann 1974; Martin and Bateson 2007).

#### Przewalski horses

Observations took place twice a day during five time slots covering the daylight period: 0700–1000, 1000–1300, 1300–1530, 1530–1800 and 1800–2100 hours. Sampling duration at each time slot was counterbalanced within and between subjects. Each focal horse was observed for 10-min sampling sessions during which all behaviours were continuously recorded. The total time of observation per subject was 600 min (10 h) performed by three trained observers who trained together until 97 % agreement was reached.

#### Domestic horses

Sampling sessions were conducted during daylight at three time slots: 0600–1200, 1200–1600 and 1600–1930 h. Horses were randomly assigned to observations on the first day, and then the observation of a given horse changed every day following a rotation schedule (thus, if one horse was observed from e.g. 0600 to 0605 hours on day 1, it was observed between 0605 and 0610 hours on day 2 and so on). All behaviours of the focal animal were recorded continuously by a single trained observer during 5-min sessions. Only one horse was observed at a time (i.e. one focal animal). The total time of observation per subject was 250 min (4.16 h).

#### Both species

All behaviours were recorded (see e.g. Waring 2003 for a detailed ethogram), but we report here only the behaviours of interest for this study. These behaviours were yawning (as defined for humans: mouth opening, deep inspiration, followed by a brief apnoea and a slow expiration; Walusinski and Deputte 2004) and, following Waring (2003) and McDonnell and Haviland (1995), aggressive (threats to bite, bite, threats to kick, kicks, chases), defensive (avoid, flee) and affiliative (olfactory investigation and approach, mutual grooming, head-body contacts) social interactions (Table 1). Both aggressive and defensive interactions were an agonistic behaviour used as a proxy of social conflicts.

**Table 1:** Social interactions recorded, adapted from Waring (2003) and McDonnell and Haviland (1995)

Agonistic aggressive	Threats to bite, bite	*Threat to bite*: laying back of the ears, the horse displays a head swing with slightly opened mouth, or it consists of a nipping motion toward an opponent using an extended head as well as neck. Physical contact is attempted while *biting*
	Threats to kick, kick	*Threat to kick*: laying back of the ears, the horse shifts the hindquarters toward the opponent. Vigorous tail switching and even slight hopping motions with the hindquarters may occur prior to kick threats. Physical contact is attempted while *kicking*
	Chase	One horse pursuits another, usually at a gallop in an apparent attempt to overtake, change the movement of or catch up with another horse
Agonistic defensive		The horse maintains or increases the distance between self and a threatening opponent: *avoiding* by moving a part of the body away, *moving away* by walking or *fleeing* by trotting or cantering
Affiliative	Approach	The horse reduces the distance between self and (the) other horse(s), ears forward
	Olfactory investigation	The horse displays nasal investigation at the other’s body (e.g. naso-nasal, flank region)
	Mutual grooming	The two partners usually face each other, standing so that one shoulder is close to the corresponding shoulder of the partner. After introductory sniffing, the grooming activity usually begins along the crest of the neck; it may then proceed to the withers, the shoulder or along the back to the croup and base of the tail
	Head-body contact	The horse places its head on the other horses’ body (e.g. back, croup)

### Statistical analyses

The horses were classified into six groups, according to species, sex and social status as regulated by sexual maturity (adult/immature) and group type (harem/bachelors). Adult intact males in the male-only group were classified as bachelors, intact males in harems of less than 1 year old as colts, females of less than 2 years old as fillies, adult intact males in harems as stallions, adult mature females as mares and mature castrated males as geldings.

All behavioural variables are presented in frequency per hour of observation. Due to lack of normality of distribution, the effect of sex and social status (investigated in both PH and DH groups) and species on yawning frequency (YF) and affiliative, defensive and aggressive interactions was evaluated by a non-parametric test (Wilcoxon two-sample test, two-sided, PROC NPAR1WAY). The *P* level was set on 0.05; however, in the case of *P* <0.1 (tendency level), the results were also presented if they seemed to be interesting. The relationship between yawning and social interactions was studied with Spearman correlations (PROC CORR). The SAS 9.3 statistical package (SAS Institute, Inc., Cary, NC) was used for the analyses. The data in text are presented as medians and quartiles.

## Results

The descriptive statistics of behavioural variables observed in both species are shown in the Appendix Table 2.

### Przewalski horses

**Fig. 1:**
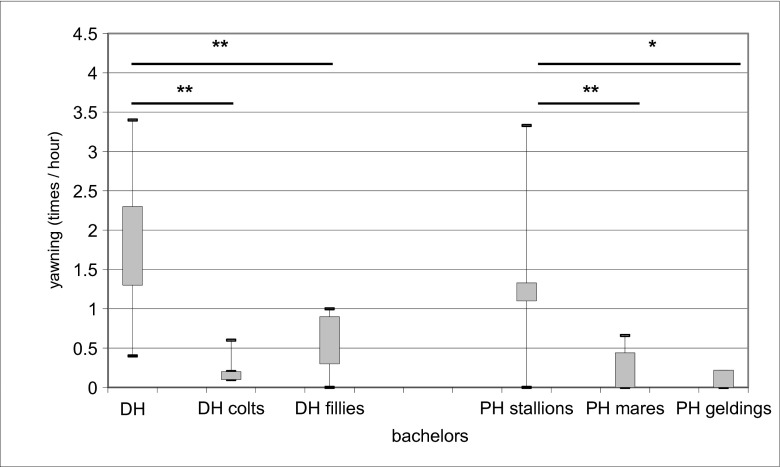
Yawning frequency in the Przewalski horse (*PH*; *Equus ferus przewalskii*) and domestic horse (*DH*: *Equus caballus*). **P < 0.05, significant differences between values; *P < 0.1, significant differences between values

The YF in PHs amounted to 0.94 ± 0.89 (Me = 0.6 [0.2; 1.3]) yawns per h, revealing high individual variations. These variations are partly due to sex and age: the adult bachelors (*N* = 8) yawned significantly more often (Me = 1.50 [1.05; 2.20] times/h) than the younger individuals of both sexes: female (0.50 [0.30; 0.90] time/h, *z* = 2.33, *P* = 0.037) and male (0.50 [0.30; 0.90] time/h, *N*_1_ = 6, *N*_2_ = 5, *z* = 2.37, *P* = 0.035) yearlings (Fig. 1).

**Fig. 2:**
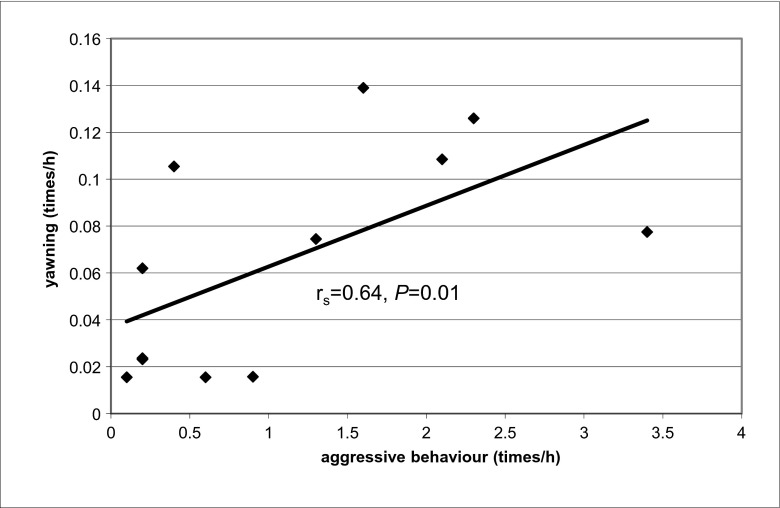
Spearman correlation between the frequency of yawning and aggressive interactions in male PHs. Black diamonds (♦) represent yawning frequency in relation to aggressive interactions occurence in individual horses

The affiliative behaviours occurred 8.7 [8.0; 12.0] times/h, whereas the defensive behaviour was observed to occur with the frequency of 0.11 [0.08; 0.16] time/h in DHs. The aggressive behaviour was displayed 0.05 [0.02; 0.07] time/h. The frequency of affiliative and defensive interactions did not differ between groups of PH. Overall, YF correlated with the frequency of aggressive behaviours (*r*_s_ = 0.59, *P* = 0.008). This correlation could be due to the tendency of more frequent aggressive behaviours in bachelors (0.09 [0.06; 0.12] compared to fillies (0.03 [0.24; 0.05], *z* = 2.13, *P* = 0.053)) and colts (0.02 [0.02; 0.03], *z* = 2.13, *P* = 0.053). This high correlation between yawning and aggressive behaviours was maintained when only the males were considered (*r*_s_ = 0.64, *P* = 0.018, Fig. 2). No other correlations were found between yawning frequency and other social behaviours.

### Domestic horses

The yawning frequency in DHs was 0.68 ± 0.86 (0.44 [0.00; 1.11]) yawns per h, revealing again high individual variations. The adult stallions yawned more (1.22 [1.11; 1.33]) than the adult females (0.33 [0.00; 0.44], *N*_1_ = 6, *N*_2_ = 10, *z* = 2.38, *P* = 0.05, Fig. 1) and tended (probably due to small sample size) to yawn more than the geldings (0.00 [0.00; 0.22], *N* = 3, *z* = 2.18, *P* = 0.062).

There was no difference either between sexes or between groups in the frequency of affiliative and agonistic interactions, whichever aggressive or defensive, but overall, the frequency of agonistic behaviours was very low (less than 0.025 time per h). There was an overall positive correlation between yawning frequency and affiliative interactions with other group members (*r*_s_ = 0.59, *P* = 0.016), but none with other social interactions which, given the low level of agonistic interactions, is not surprising.

### Comparison between species

**Fig. 3:**
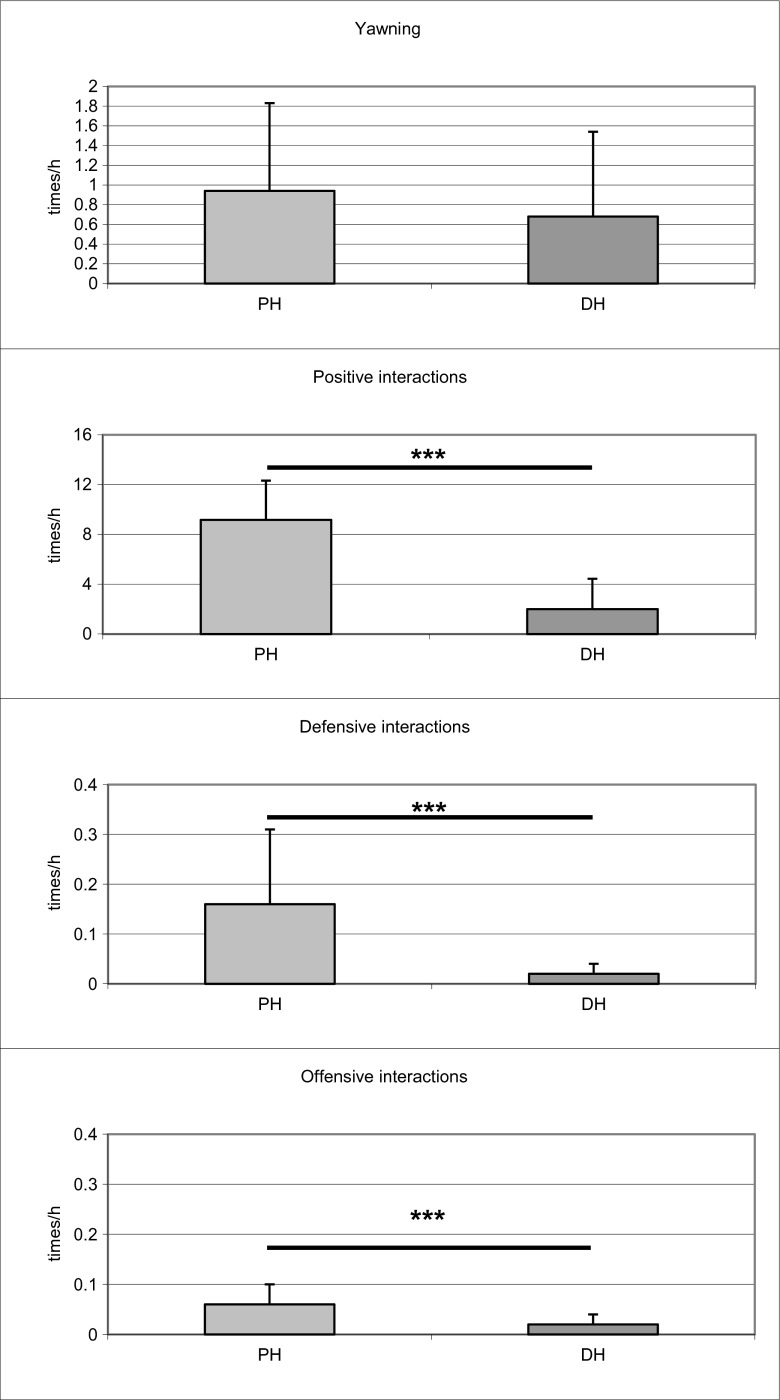
Yawning, affiliative interaction and agonistic interaction in the Przewalski horse (*PH*; *Equus ferus przewalskii*) versus domestic horse (*DH*: *Equus caballus*). **P < 0.05, significant differences between values; ***P < 0.01, significant differences between values

Przewalski and domestic horses did not differ in YF, both at the group or population levels (Fig. 3). However, PHs, when compared to DHs, were characterised with higher occurrences of social interactions overall: affiliative (8.7 [9.0; 12.0] and 0.66 [0.22; 5.88], *N*_1_ = 19, *N*_2_ = 19, *z* = 5.18, *P* < 0.001), defensive (0.11 [0.08; 0.16] and 0.01 [0.00; 0.06], *z* = 5.47, *P* < 0.001) and aggressive (0.03 [0.02; 0.07] and 0.20 [0.00; 0.03], *z* = 3.01, *P* < 0.001) behaviours.

In particular, the PH bachelors were engaged in more affiliative (8.35 [7.30; 10.30] times/h), defensive (0.11 [0.10; 0.17] time/h) and aggressive (0.09 [0.06; 0.12] time/h) interactions than domestic stallions (4.33 [0.22; 6.44] times/h, *N*_1_ = 8, *N*_2_ = 6, *z* = 4.72, *P* = 0.003; 0.01 [0.00; 0.01] time/h, *z* = 2.52, *P* < 0.001; and 0.02 [0.00; 0.02] time/h, *z* = −3.38, *P* < 0.001, for affiliative, defensive and aggressive behaviours, respectively).

## Discussion

The present study, where social groups of both wild and domestic horses were observed, reveals for the first time that in these species, as in other species studied, adult males yawn more than females and immature males. It also shows that in natural conditions, yawning is a rather rare behaviour in mares and geldings with 0.07 occurrences per h, whether wild or domestic horse species being considered. However, when looking only at adult males of both species, yawning frequencies were comparable to the frequencies observed for the domestic mares and geldings kept in restricted living conditions (Fureix et al. 2011).

The frequency of social interactions was higher in Przewalski horses, especially in bachelors, as mentioned in earlier studies (Feh 1988; Christensen et al. 2002) which may explain that the unequivocal relationship between agonistic behaviour and yawning frequency was found only in male Przewalski horses. However, the correlation found between yawning frequency and affiliative social interactions in the domestic horses indicates that yawning may, to some extent, relate to social (probably excitatory) contexts. Further studies are needed to look more precisely at which aspects of the social interactions may be involved (for example, sniffing may precede different types of interactions of affiliative or negative valence) and whether the structure of yawning may change accordingly.

### Domesticated versus wild species: domestication and impact of life conditions

The absence of species differences in yawning frequency clearly indicates that domestication did not influence the prevalence of this behaviour. However, the study of Fureix et al. (2011) indicated very high frequencies of yawning as compared to our findings here: 0.02 per min in one site where only geldings were present and 0.05 per min in another site where both geldings and mares were observed. Compared to the frequencies observed for the domestic mares and geldings in the present study, these frequencies are about 60 times higher. Since in the current study the comparison of Przewalski and domestic horses living in stable groups in semi-natural conditions did not indicate species differences, it is likely that the increased frequencies of this behaviour in the domestic situation rather reflects an impact of the housing conditions (see also findings on other *ambiguous* behaviours in Hausberger et al. 2012; Blois-Heulin et al. 2015) than of domestication itself. Indeed, the conditions of life between both studies were drastically different as Fureix et al. (2011) observed the horses in riding centres where they were housed in single stalls, thus in restricted social, spatial and feeding conditions known to elicit chronic stress (McGreevy et al. 1995). These horses were working in riding lessons, which may also be a source of discomfort (e.g. Lesimple et al. 2010). The finding that yawning frequency co-occurred with stereotypic behaviours, an admitted indicator of present and/or past exposure to stressors reinforces the idea that this behaviour was potentially triggered by chronic stress (Fureix et al. 2011). Interestingly, the frequency of yawning and stereotypic behaviours increased in the pre-feeding situation, which likely corresponds to anticipation and potential frustration, and, therefore, an excitatory situation. Anticipatory behaviours likely correspond to high-intensity emotions (whether positive or negative), and the results obtained in the present study may indicate that yawning is associated with such excitatory states whatever their valence (Mendl et al. 2010; Peters et al. 2012). It could be argued that the high level of excitability could be attributed to mental characteristics of horses (e.g. due to breed, Hausberger et al. 2004; Lloyd et al. 2008) from the cited study, but almost a half of the domestic horses observed were also warmbloods which did not show higher yawning frequencies than the more native Konik polski horses. Excitation may thus also trigger this behaviour. Stress yawn (*yawning-stretching syndrome*, Gessa et al. 1966) was also observed out of social context but in different anticipatory situations such as highly aroused dogs before a walk (Górecka-Bruzda, personal observations) and before feeding in lions and mandrills (Baenninger 1997).

### Yawning and social stress

Prevalent occurrence of yawning in primates was observed primarily in social conflict situations (Hall 1962; Hinde and Rowell 1962; Hadidian 1980; Troisi et al. 1990; Maestripieri et al. 1992); therefore, social tension was hypothesised to be the cause of *stress yawns* in primates (Deputte 1978). Other arousal-provoking situations like feeding competition were also observed to be a context where subordinate horses yawn more often (Hausberger, personal observations).

Here, we used agonistic behaviour frequency as an indicator of social conflicts and looked at the associated yawning frequency. We found that male Przewalski horses (bachelors) were more engaged in all measured social interactions than younger individuals, corroborating previous reports of increased activity in adult male horses in natural conditions (Duncan 1980; Berger 1986). According to previous results in this species (Bourjade et al. 2009), PH bachelors performed overall more affiliative interactions than agonistic ones. However, bachelors involved in social conflicts, exhibiting a more aggressive behaviour than others, also showed increased yawning frequencies. This result would support the *social stress hypothesis* proposed for primates’ yawning behaviour. It is puzzling yet that domestic horses did not behave the same way; we found no correlation between yawning frequency and aggressive or defensive interaction frequencies in domestic horses, which may simply be due to the very low frequency of agonistic behaviours observed in these conditions. Since there were less adult males in domestic horse groups than in Przewalski horse groups, it might be possible that the small number of conflicts observed have hampered the association between yawning frequency and agonistic behaviour in domestic horses being revealed.

There was, however, a positive correlation between yawning frequency and affiliative behaviour in domestic horses. It would be interesting to examine more precisely which positive interactions were most involved with yawning and what followed in terms of social issue. In this respect, it is worth noting that several contexts may be responsible for the change of the arousal state, including its increase or decrease, depending on trigger’s valence (positive or negative). The yawning, as primarily connected with relaxation and increased drowsiness (Walusinski and Deputte 2004; Guggisberg et al. 2010) may be, as suggested by Deputte (1994), associated with neural mechanisms of lowering arousal level.

### Sex differences

Here, it was remarkable that despite the species difference, but also the differences in group composition, the same overall frequency of yawning appeared to be higher for adult intact males than for females or immature horses in all cases.

In rats (Berendsen and Nickolson 1981; Serra et al. 1984; Urbá-Holmgren et al. 1990) and primates (Troisi et al. 1990; Walusinski and Deputte 2004), yawning was found to be more frequent in males than in females. Sex difference in yawning frequency was observed mostly in primate males of *Cercocebus albigena* and *Macaca fascicularis* (Deputte 1978; Troisi et al. 1990), *Macaca mulatta* (Chambers and Phoenix 1981; Deputte et al. 1994) *Macaca fuscata* (Troisi et al. 1990) and *Macaca nigra* (Hadidian 1980), usually after reaching sexual maturity. It can be admitted that the testosterone level was involved in the regulation of yawning in the species observed. In rats, Berendsen and Nickolson (1981) found that castration reduced the frequency of yawning in males, but not in females. In the latter study, testosterone treatments increased the number of yawns in castrated males and in both intact and ovariectomised females, suggesting that yawning is under androgenic control. Experiments on apomorhine’s (non-selective dopamine agonist) effects in rats have shown a higher increase of yawning frequency in male than in female (Serra et al. 1984). The administration of 17β-oestradiol reduced the effect of apomorhine-induced yawning in male rats (Serra et al. 1984) which suggests that sex hormones, mutually interdependent, are involved in the regulation of yawning in animals. Interestingly, the study of Fureix et al. (2011), performed on females and castrated males, mentioned no sex difference in yawning frequency. This could be due to the low testosterone levels in geldings since stallions were not available for the latter study. The geldings observed in the semi-natural conditions of the present study also tended to show lower frequencies of yawning than stallions (or even mares, although not significant), suggesting a relation between hormonal levels and this behaviour also in the two herbivore species studied here. The observation of higher frequencies of yawning in mature stallions as compared to immature males further promotes this idea.

## Conclusions

According to existing works, yawning may be triggered by different factors like higher level of testosterone and, simultaneously or independently, by social stress caused by agonistic interactions, mainly between males. In the present study on horses living in favourable environment close to natural settings, it seems, however, that testosterone may be a major factor, while social excitation (rather than social stress) appears as a secondary potential factor causing a higher frequency of yawning in males, because they yawned also when not involved in social conflicts. Since a high frequency of yawning was related to increased frustration in horses kept in a restricted stabling environment (Fureix et al. 2011), it may also be supposed that the lower frequency of yawning in horses observed in undisturbed social groups may reflect increased welfare in equine groups living in favourable conditions satisfying their behavioural needs. Increased occurrence of yawning in domestic situations could thus attract the attention of caretakers to make the alterations to improve the welfare of their horses.

While the precise relationship between the causes, contexts and functionality of yawning remains to be explained, this study contributes to the general knowledge on this behavioural pattern in herbivores.

## Appendix

**Table 2:** Descriptive statistics of behavioural variables in the Przewalski horse (*Equus ferus przewalskii*) and domestic horse (*Equus caballus*)

Species/group	Yawning		Affiliative behaviour		Defensive interactions		Aggressive interactions	
	Mean ± SD	Median [Q1; Q3]	Mean ± SD	Median [Q1; Q3]	Mean ± SD	Median [Q1; Q3]	Mean ± SD	Median [Q1; Q3]
Przewalski horses	0.99 ± 0.89	0.60 [0.20; 1.30]	9.70 ± 2.99	8.70 [8.00; 12.00]	0.16 ± 0.14	0.11 [0.08; 0.16]	0.05 ± 0.04	0.03 [0.02; 0.07]
Bachelors^a^, *N* = 8	1.66 ± 0.94	1.50 [1.05; 2.20]	8.66 ± 2.89	8.35 [7.30; 10.30]	0.19 ± 0.21	0.11 [0.10; 0.17]	0.09 ± 0.04	0.09 [0.06; 0.12]
Colts^b^, *N* = 5	0.53 ± 0.38	0.50 [0.30; 0.90]	9.84 ± 4.56	12.2 [9.3; 12.6]	0.18 ± 0.10	0.16 [0.13; 0.21]	0.03 ± 0.02	0.02 [0.02; 0.03]
Fillies^c^, *N* = 6	0.53 ± 0.38	0.50 [0.30; 0.90]	9.28 ± 2.52	8.40 [7.40; 10.70]	0.10 ± 0.04	0.0 [0.07; 0.14]	0.04 ± 0.01	0.03 [0.24; 0.05]
Domestic horses	0.63 ± 0.82	0.44 [0.00; 1.11]	1.86 ± 2.26	0.66 [0.22; 5.88]	0.02 ± 0.02	0.01 [0.00; 0.06]	0.02 ± 0.02	0.02 [0.00; 0.03]
Males^d^, *N* = 6	1.37 ± 1.08	1.22 [1.11; 1.33]	3.67 ± 2.94	4.33 [0.22; 6.44]	0.02 ± 0.02	0.01 [0.00; 0.00]	0.01 ± 0.01	0.02 [0.00; 0.02]
Mares^e^, *N* = 10	0.27 ± 0.25	0.33 [0.00; 0.44]	0.64 ± 0.79	0.33 [0.22; 0.88]	0.01 ± 1.93	0.01 [0.00; 0.02]	0.03 ± 0.02	0.02 [0.01;0.04]
Geldings^f^, *N* = 3	0.07 ± 0.12	0.0 [0.0; 0.22]	1.19 ± 0.68	1.33 [0.44; 1.77]	0.03 ± 0.03	0.02 [0.08; 0.06]	0.01 ± 0.01	0.02 [0.08; 0.06]

^a^Adult intact males in the male-only group

^b^Intact males of less than 1 year old

^c^Females of less than 2 years old

^d^Sexually mature males

^e^Sexually mature females

^f^Castrated males
